# Review: Traumatic brain injury and hyperglycemia, a potentially modifiable risk factor

**DOI:** 10.18632/oncotarget.11958

**Published:** 2016-09-10

**Authors:** Jia Shi, Bo Dong, Yumin Mao, Wei Guan, Jiachao Cao, Rongxing Zhu, Suinuan Wang

**Affiliations:** ^1^ Department of Neurosurgery, The Third Affiliated Hospital of Soochow University, Changzhou, China

**Keywords:** hyperglycemia, traumatic brain injury, blood glucose, glycemic control

## Abstract

Hyperglycemia after severe traumatic brain injury (TBI) occurs frequently and is associated with poor clinical outcome and increased mortality. In this review, we highlight the mechanisms that lead to hyperglycemia and discuss how they may contribute to poor outcomes in patients with severe TBI. Moreover, we systematically review the proper management of hyperglycemia after TBI, covering topics such as nutritional support, glucose control, moderated hypothermia, naloxone, and mannitol treatment. However, to date, an optimal and safe glycemic target range has not been determined, and may not be safe to implement among TBI patients. Therefore, there is a mandate to explore a reasonable glycemic target range that can facilitate recovery after severe TBI.

## INTRODUCTION

Traumatic brain injury (TBI), is a leading cause of death among adults [1]. The term TBI refers to a diverse group of head injuries in which a patient receives a Glasgow Coma Scale (GCS) score between 3 and 8. Much progress has been made in the prevention and treatment of TBI over the last 20 years yet nearly 2 million people sustain a TBI annually in the United States, contributing to one-third of all injury-related deaths [2]. In general, the damage induced by TBI is divided into two types: primary and secondary brain injuries. Primary injury is initiated by a physical insult to the head, resulting in compression and shearing of adjacent tissues with or without loss of consciousness. Secondary injury is a complex process that occurs in the hours and days following primary injury, which encompass cranial and systemic complications. Cranial complications may include cerebral edema [3], intracranial hypertension [4], infection [5], calcium ion toxicity [6] and vasospasm [7]. Systemic complications are mainly ischemic in nature [8], and include hypoxemia[8][9], hypotension, hypertension [8], hyperglycemia, and hypoglycemia [10]. Among these secondary complications, hyperglycemia (both peak glucose and persistent hyperglycemia) in TBI patients is one of the most common and correlates with the severity of the injury and clinical outcome [11, 12]. Furthermore, the link between hyperglycemia and poor prognosis is also observed in ischemic stroke, subarachnoid hemorrhage, and intracerebral hemorrhage [13–15]. Importantly, as treatments for hyperglycemia become available, blood glucose control has attracted significant attention as a promising intervention for reducing the complications of TBI.

Previous studies have demonstrated that hyperglycemia plays a causative role in promoting poor clinical outcomes in TBI. In this review, we summarize the potential causes and effects of hyperglycemia in TBI patients and attempt to address the pathological mechanisms that might explain the association of hyperglycemia with poor clinical outcomes. Furthermore, we perform a systematic review of research studying glycemic control in TBI patients and provide some direction for future clinical prevention and treatment.

## THE BRAIN AND GLUCOSE (FIGURE 1)

Glucose is the predominant source of energy for the mammalian brain [16]. Glucose entry into the brain is facilitated by the transporter, GLUT1, which is found in the blood-brain barrier (BBB). GLUT1 mediates glucose uptake from extracellular fluid into astrocytes, microglia and oligodendroglia, whereas transporter GLUT3 facilitates neuronal glucose uptake [17, 18]. Compared to other cells in the adult brain, neurons and astrocytes have a stronger demand for glucose. However, the underlying relationships between neuronal and astrocytic glucose utilization have remained controversial for decades because current technologies do not possess adequate resolution to quantify metabolic activity in a single cell *in vivo* [17, 19, 20].

To date, there are two conflicting theories describing the cellular fate of glucose, and which propose different directions for lactate shuttling between neurons and astrocytes. These are termed the astrocyte-neuron lactate shuttle (ANLS) and the neuron-astrocyte lactate shuttle (NALS). The ANLS theory proposes that glucose is transported into astrocytes through the GLUT1 transporter and then metabolized to lactate [21, 22]. Lactate is transported to the outside of astrocytes and taken up by neurons and served as fuel through monocarboxylate transporters (MCTs) [23]. The NALS model predicts that neurons uptake glucose during activation and then transfer lactate to astrocytes [24]. Interestingly, if glucose and lactate are both present, neurons appear to have a preference for lactate [23]. Both neurons and astrocytes have strong demands for energy and are largely intolerant of inadequacy of energy supplies. Therefore, disruption of glucose shuttling and/or metabolism may result in various central nervous systemic pathologies [25, 26], and it may be important to rigorously define the relationship between glucose metabolism in brain cells and cerebral diseases.

**Figure 1:**
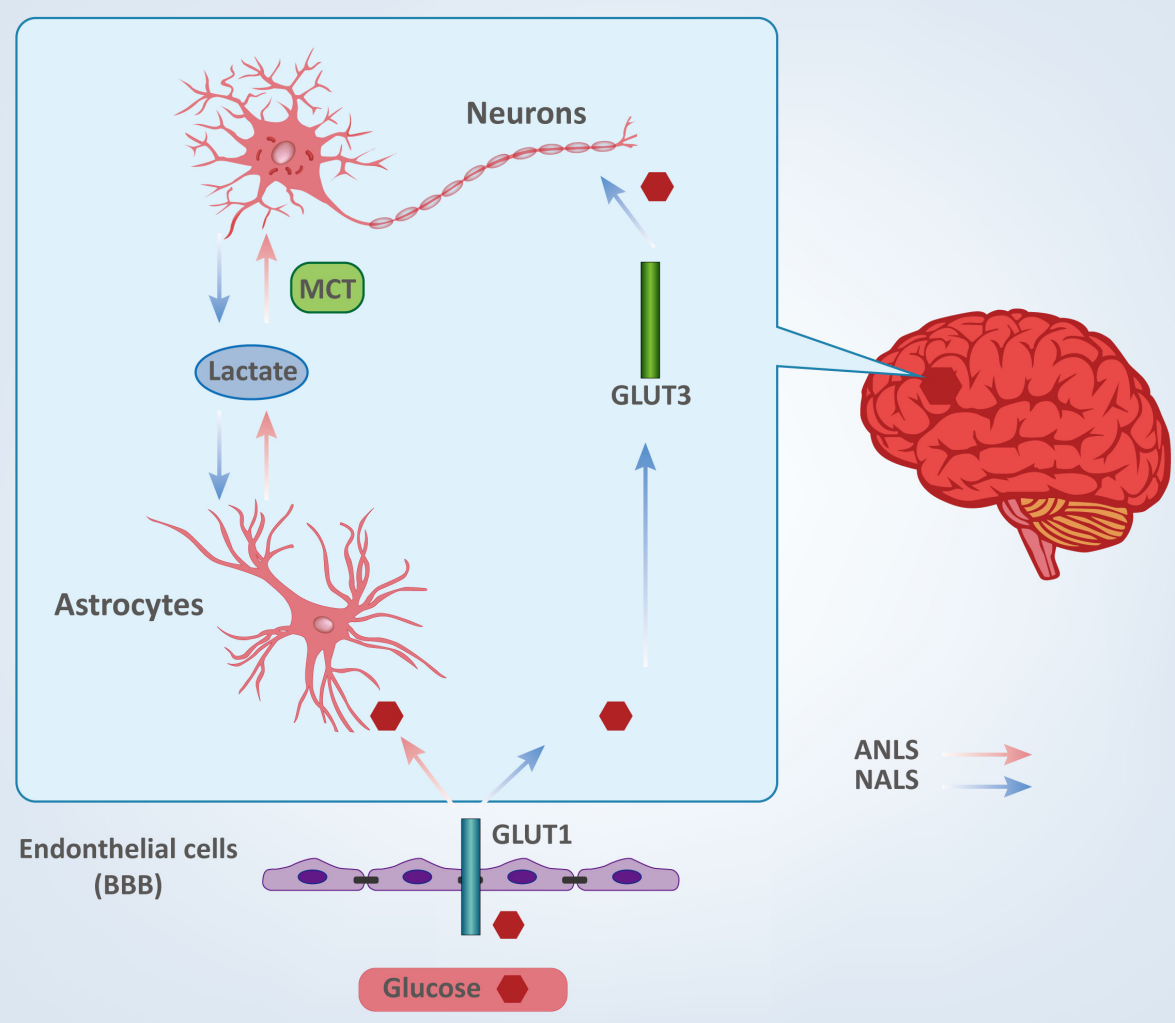
Cellular fate of glucose between neurons and astrocytes

## MECHANISMS OF HYPERGLYCEMIA AFTER TRAUMATIC BRAIN INJURY (FIGURE 2)

In a 4-year study of 149 patients with TBI, those with severe TBI had greater admission blood glucose levels compared to those with mild TBI [27]. This initial hyperglycemia was associated with increased mortality (37% in the hyperglycemia group versus 8% in the normoglycemia group), indicating a close relationship between TBI and glucose levels [28]. Thus, exploring the underlying causes leading to hyperglycemia after TBI may provide new treatment options. The following sections address a number of mechanisms by which hyperglycemia is induced following TBI.

### Stress response

There are several explanations for high glucose levels after TBI, and one of the most studied is stress-induced hyperglycemia. During a 4-year study, a total of 626 patients were enrolled with severe TBI defined by GCS score from 3 to 8. Among 184 patients admitted with hyperglycemia, 152 (82.6%) were diagnosed with stress-induced hyperglycemia (SIH) and 32 patients (17.4%) were diagnosed with diabetic hyperglycemia (DH). Patients with SIH had a 50% increased mortality as compared to nondiabetic hyperglycemia patients, whereas DH patients did not have a significant increase in mortality [29]. During TBI, the hypothalamic-pituitary-adrenal axis and the sympathetic autonomic nervous system are activated, resulting in increased levels of neuro-hormonal factors and insulin resistance.

First, the increased levels of stress hormones may influence glycemic levels. After TBI, the activation of the hypothalamic-pituitary-adrenal axis and the sympathetic autonomic nervous system induce elevated blood levels of catecholamine, cortisol, glucagon and growth hormone, which enhance glycogenolysis and hypermetabolism and lead to excessive glucose production [29, 30]. By functioning on islet beta cells' alpha 2 receptor, catecholamines also increase glucagon production and decrease insulin secretion [31, 32]. Second, transient insulin resistance (IR) is another mechanism by which stress hormones may affect glucose metabolism. Several reports have demonstrated that TBI reduces insulin sensitivity and secretion, contributing to IR and glucose homeostasis impairment [33]. The released catecholamines can inhibit glucose transport *via* inhibition of insulin binding, leading to IR with hyperinsulinemia [34].

### Inflammatory response

TBI is accompanied by a systemic inflammatory response syndrome (SIRS), in which there may be two mechanisms that lead to hyperglycemia. First, during the inflammatory response, several cytokines are released, including TNF-α, IL-6, and CD11d [35]. Studies demonstrated that TNF-α regulated quantity and function of glucose by decreasing levels of adipocyte-specific genes and increasing levels of preadipocyte-specific genes, contributing to insulin resistance and hyperglycemia [36]. Second, inflammatory responses increase the level of corticotrophin-releasing hormone (CRH) and stimulate the release of adrenocorticotropic hormone (ACTH) from the anterior pituitary, which induce elevated levels of blood glucose. Nitric oxide (NO), which is activated in the inflammatory response, participates in signal transduction pathways that lead to the release of corticosterone from the adrenal gland, and finally results in hyperglycemia. [37]. While not still understood, the interaction between inflammatory response and hyperglycemia is clearly important and warrants further investigation.

### Diabetes mellitus (DM)

Diabetes mellitus (DM) is another source of hyperglycemia in this setting. Severe TBI with DM has a higher mortality (14%) compared to severe TBI without DM (8.2%), and TBI with insulin-dependent DM (IDDM) has a higher mortality rate (17.1%) than patients without insulin-dependent DM (NIDDM). These results indicate that insulin deficiency may contribute to the increased mortality after TBI, and DM has the potential to be an independent predictor of poor outcome and mortality after TBI [38, 39]. Latent diabetes mellitus may be particularly problematic, especially in elderly patients. The symptoms of DM can be exacerbated after severe TBI, and undiagnosed DM may contribute to insulin resistance without characteristic clinical symptoms [40].

### Pituitary and hypothalamic dysfunction

Pituitary and/or hypothalamic dysfunction following TBI is a significant occurrence that is rarely considered by clinicians. Recently, this topic has attracted some attention owing to high prevalence of pituitary or hypothalamic dysfunction after moderate-severe TBI and its association with increased morbidity and poor recovery [41]. In addition to its role in regulating the stress response, the hypothalamus-pituitary axis is vital for balancing glucose homeostasis, acting to decrease hepatic gluconeogenesis and increase insulin sensitivity [42]. Pituitary dysfunction directly leads to impaired glucose metabolism and may act as a potential cause of hyperglycemia [43]. In addition, injury of the pituitary leads to disorders of neuroendocrine function [44, 45], and, amongst these, deficiency of gonadotrophic hormones and growth hormones are commonly induced by TBI and may result in an imbalance of glucose metabolism [46]. Thus, pituitary/hypothalamic dysfunction may contribute significantly to hyperglycemia in TBI patients.

### Iatrogenic factors

There are several other explanations of hyperglycemia induced by TBI. Surgery,anaesthesia, treatment strategies (including too high a dosage of hyperosmotic glucose or a high calorie substance), and psychological factors, may also lead to hyperglycemia in TBI patients, especially in severe TBI [47, 48].

In conclusion, an elevated stress and inflammatory response seem to be the major causes of hyperglycemia after TBI. Preexisting hyperglycemia does not seem to have a predominant role in TBI patients, and a relationship between pituitary and/or hypothalamic dysfunction, iatrogenic factors, and hyperglycemia is plausible.

## HYPERGLYCEMIA AND CLINICAL OUTCOMES AFTER TRAUMATIC BRAIN INJURY

**Figure 2:**
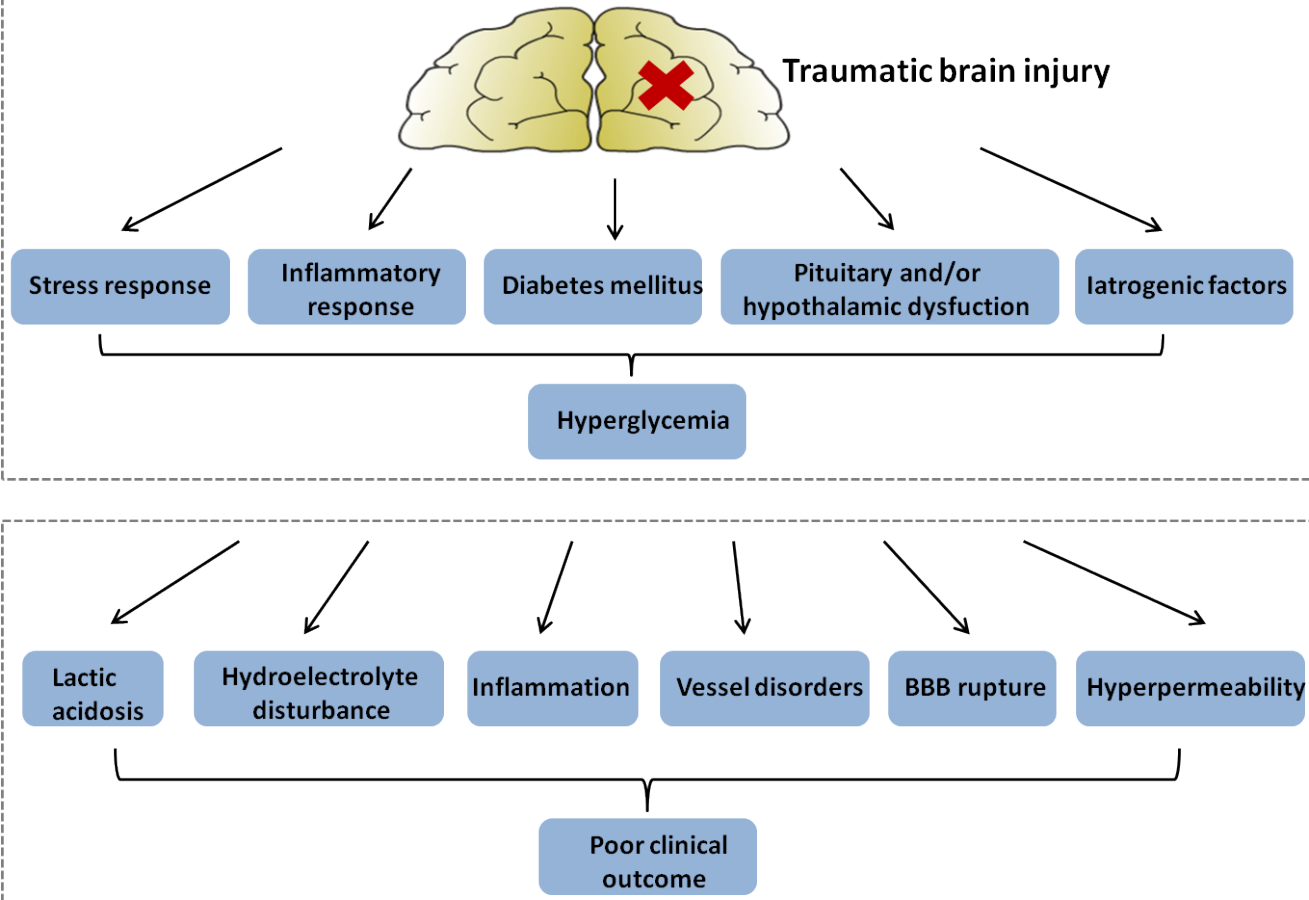
Figure illustrating hypothetical mechanisms: hyperglycemia in patients with traumatic brain injury (upper panel); and explaining a detrimental effect of hyperglycemia on clinical outcome (lower panel) BBB, blood-brain barrier.

During one study, a total of 267 patients with moderate or severe TBI (GCS scores 3-12) were admitted. Patients with severe TBI had significantly higher glucose levels than those with moderate injury. Furthermore, patients who subsequently had an unfavorable outcome had higher glucose levels (>200 mg/dL or>11mmol/L) than did those with a better prognosis [11]. In addition, the relationship between high blood glucose levels and poor outcome after TBI appears to be more pronounced with persistent hyperglycemia (PH) than solely hyperglycemia on admission [49]. One study showed that PH was an independent predictor of clinical outcome in severe TBI, and PH was associated with higher mortality rates in patients with severe TBI [50]. These studies beg the question: How exactly does hyperglycemia affect outcomes after traumatic brain injury? (Figure 2) Here we show that hyperglycemia may increase the damage of nerve function and brain structure in a number of ways.

### Lactic acidosis

In the early stages after severe TBI, local blood flow decreases [51]. During ischemic and hypoxic conditions, hyperglycemia inhibits the tricarboxylic acid cycle (TCA) and promotes anaerobic glycolysis, contributing to the accumulation of lactic acid and disturbance of pH homeostasis. In support of a detrimental function of lactic acid is the discovery that in patients with severe TBI, hyperglycemia is associated with both intracellular acidosis and disruption of BBB, eventually leading to ischemia, edema and necrosis [52, 53]. Additionally, injured nerve cells cannot metabolize the excess glucose using the mitochondrial aerobic metabolic pathway, causing general decreased glucose utilization [54]. Finally, severe TBI or a reduced blood supply leads to increased metabolism of local brain tissues further stimulating glycolysis [55]. The above pathways promote accumulation of lactic acid and eventually contribute to dysfunctional energy metabolism in nerve cells.

### Electrolyte disturbances

Within minutes after TBI, dysfunctional energy metabolism and lactic acidosis lead to increased intracellular Ca^2+^, causing cellular toxicity and the accumulation of free fatty acid (FFA) and glutamate [56, 57]. Excessive glutamate promotes increased Na^+^ and Ca^2+^ to enter into cells, and the resulting intracellular Ca^2+^ overload stimulates cell damage *via* caspase activation [58]. As astrocytes represent the main defense by absorbing glutamate, an initial glutamate excitotoxicity occurs in neurons [59]. However, during the secondary injury stage, if extracellular glutamate levels are constantly increased, the expression of glutamate transporters (glutamate transporter-1, GLT-1) and the glutamate-aspartate transporter (GLAST) decreases, and the intracellular Na^+^ can reverse transport. This event favors the release of glutamate from astrocytes rather than neurons, and the astrocytes actually enhance glutamate excitotoxicity [60, 61]. Additionally, a relatively large amount of lactic acid leads to reduced ATP and impairs the Na^+^-K^+^-ATPase causing excessive extracellular Na^+^ levels and cell edema that ultimately results in intracranial hypertension and cerebral hernia [62].

### Inflammation

Hyperglycemia after severe TBI is associated with an increase in pro-inflammatory cytokines and pro-inflammatory transcription factors, such as transforming growth factor (TGF-β), interleukin 1β (IL1-β) and tumor necrosis factor-α (TNFα) [63, 64]. These cytokines increase significantly in the peripheral blood, cerebrospinal fluid, and brain tissue after TBI, which closely correlates with the severity and bad outcome of TBI patients [65]. An *in vitro* experiment demonstrated that hyperglycemia induced by walnut oil increased the release of pro-inflammatory cytokines, which led to reduced antioxidant capacity and oxidative DNA damage of human monocytic cells (U937) [66]. Furthermore, microglia activation and endothelial cell damage induced by inflammation after TBI could lead to brain injuries, such as edema [67]. Therefore, cytokines play an important role in the neuropathology of TBI, and inhibition of these cytokines indeed had a correlation with neuroprotective effects after TBI, especially the improvement of brain edema and neurological outcomes [68].

### Other effects

Hyperglycemia can affect clinical outcomes in other ways. For instance, hyperglycemia can directly cause increased blood viscosity and diffuse small vessel disorders, resulting in ischemia and hypoxia in the brain tissues [69]. Additionally, hyperglycemia exacerbates edema of endothelial cells, impairs glial cells, and can induce the rupture of the BBB [70, 71]. Hyperglycemia also has the potential to accelerate cerebral vasospasm [72]. Thus, the causal relationship between hyperglycemia and poor clinical outcome in TBI patients may be multifactorial. In the sequence of insults that occur after TBI, hyperglycemia may play a detrimental role by elevating secondary complications such as dysregulated energy metabolism, infection, intracranial edema, and intracranial hypertension.

## MANAGEMENT OF HYPERGLYCEMIA IN PATIENTS WITH TRAUMATIC BRAIN INJURY

As discussed, hyperglycemia may manifest poor outcomes after TBI through several responses. Besides lowering blood glucose with insulin, there are additional strategies clinicians can use to normalize glucose homeostasis that are potentially relevant in this setting. Current thinking suggests that comprehensive measures should be taken to address hyperglycemia after TBI.

### Nutritional support

Patients with severe TBI are usually in catabolic and hyperglycemic states, and may have gastrointestinal dysfunction [73]. Previous studies have shown that malnutrition is associated with a higher mortality rate in severe TBI patients, and early effective nutritional support may improve insulin resistance and patient prognosis [74]. Furthermore, studies have demonstrated that enteral feeding has greater advantages than parenteral nutrition (PN), which may be attributed to the following: 1) enteral feeding is relatively safe, cheap, cost-effective, and more physiologic; 2) enteral feeding stimulates all gastro-intestinal tract functions;; and 3) enteral feeding preserves the immunological gut barrier function and reduces septic complications [75]. It is also worth noting that a large amount of glucose (intravenous and enteral feeding) can have harmful effects on outcome in TBI patients, thus clinicians should strictly limit the dosage of glucose, which includes both intravenous glucose and enteral feeding with low carbohydrates [76–78].

### Glucose control

Insulin increases glucose utilization and reduces the damage of hyperglycemia to brain cells [79]. Owing to insulin resistance after TBI, most studies use exogenous insulin and dynamically monitor blood glucose to fight against hyperglycemia after TBI [79]. In 2001, intensive insulin therapy (IIT) was implemented in intensive care units (ICUs) worldwide after a landmark clinical trial, which demonstrated clinical benefits of IIT in a surgical ICU [80]. However, several later trials could not confirm the findings of this study, one study demonstrated that maintaining low blood glucose with IIT (a blood glucose target of 81-108 mg/dL (4.5-6 mmol/L)) was in fact associated with an increased risk of mortality and even hypoglycemia (glucose<40 mg/dL (2.2 mmol/L)), which may be due to the effects of IIT on cerebral glucose homeostasis after severe TBI [81, 82]. Another study also showed that IIT results in a net decrease in microdialysis glucose but an increase in microdialysis glutamate and lactate/pyruvate, with an adverse effect on the long-term recovery of neurological function [83]. Therefore, in patients with severe TBI, decreased glucose levels with insulin can induce and aggravate secondary brain injury.

Because of these conflicting findings, blood glucose control in TBI has recently been the topic of much research [84]. One retrospective study enrolled a total of 228 patients suffering from severe TBI that were treated with insulin. In the first week (acute stage), a blood glucose target of 90-144 mg/dL (5-8 mmol/L) was associated with reduced mortality rate and decreased intracranial pressure (ICP) compared to a blood glucose target of 63-117 mg/dL (3.5-6.5 mmol/L). However, in the second week, the groups appeared to have the reverse outcomes: compared with the 5-8 mmol/L target group, the 3.5-6.5 mmol/L group demonstrated a decreased incidence of ICP and reduced infectious complications. Therefore, a slightly higher blood glucose (5-8 mmol/L) seems to provide benefits during the first week while lower blood glucose (3.5-6.5 mmol/L) may be more favorable during the later stages of recovery [79]. Another study demonstrated that blood glucose < 6-11 mmol/L could reduce mortality in patients with mild TBI, while in patients with severe TBI, the ideal target of blood glucose was 7.77-10.0 mmol/L. Consequently, the debate on blood glucose levels in TBI is still controversial and needs to be further explored [76].

### Other treatments

There are also other strategies that can augment glucose control in the clinical setting. First, during a randomized controlled study in patients with severe TBI, moderate hypothermia was induced within 24 hours of injury, and the hypothermic group (198 cases, rectal temperature 32.0°C to 35.0°C) was found to have a lower incidence of ICP and decreased levels of glucose compared to the control group (198 cases, rectal temperature 36.5°C to 37.0°C) [85]. Multivariate regression analysis revealed that blood glucose greater than 10 mmol/L was an independent predictor for a poor outcome after severe TBI. In this clinical study, blood glucose in the hypothermia group was 100% less than 10 mmol/L, compared to 75.6% in the normothermia group after 3 days of treatment, which may be associated with the better recovery observed in patients [86]. Second, the application of naloxone, an opioid receptor antagonist, may effectively exert anti-inflammatory effects and improve the metabolism of brain cells in the early stages of severe TBI; on the other hand, naloxone also relieves calcium overload and prevents the toxicity of excitatory amino acids [87, 88]. Third, mannitol or hypertonic saline administration are effective ways of decreasing hyperpermeability and raised ICP induced by hyperglycemia after severe TBI [89, 90].

## CONCLUSIONS

Hyperglycemia (both peak glucose and persistent hyperglycemia) is one of the most common secondary complications of severe TBI and is associated with poor clinical outcomes after this type of injury. In this review, we have summarized the mechanisms leading to hyperglycemia after TBI, including stress response, inflammatory response, diabetes mellitus, pituitary and/or hypothalamic dysfunction, surgery, and anaesthesia. TBI patients with hyperglycemia possess an increased risk of poor outcome and an increased mortality rate, which is more pronounced with persistent hyperglycemia (PH) than hyperglycemia on admission [49]. Some of the direct effects of hyperglycemia may be attributed to lactic acidosis, electrolyte disturbances, inflammation, vessel disorders, rupture of the BBB, and hyperpermeability. At present, the treatments of secondary brain injury with hyperglycemia may include nutritional support, glucose control, moderated hypothermia, naloxone, and mannitol. Although the relationship between hyperglycemia and severe TBI has made great progress in recent years, there are still many treatment limitations and gaps in our current knowledge.

One such gap is the best definition of an optimal target range of blood glucose. To date, the optimal glycemic target range is uncertain and may not be uniform among TBI patients, varying between individuals at different time points during the clinical course. Cerebral microdialysis, which is defined as a neurochemical research tool in neurointensive care, can be widely used to monitor the metabolic state of the injuried brain and discover metabolic crises established as low/high glucose level or high lactate/pyruvate ratio, and may facilitate the identification of a safe and effective glycemic target range [91, 92]. In addition, although the technology for studying brain cell metabolism has advanced significantly, research should focus on examining the relationship between brain cell metabolism and hyperglycemia after severe TBI.

There is not widespread agreement on the best way to approach glycemic control in patients with TBI. Several studies have suggested that a large scale randomized controlled trial (RCT) may provide convincing proof of glucose control in future treatments. As previously mentioned, the therapy of IIT for hyperglycemia post TBI remains controversial to date, and the treatment comes at the cost of an increased risk of hypoglycemia (glucose < 2.2 mmol/L). Therefore, methods to improve IIT without inducing secondary complications should be investigated, and attention should also focus on the prevention of hypoglycemia in severe TBI patients. Multidisciplinary teams should play a role in providing individual treatments for hyperglycemia in TBI patients, including diabetic and TBI experts to facilitate the implementation of glucose control protocols aimed at lowering blood glucose without secondary complications. During the clinical course, close monitoring of blood glucose and frequent adjustment of insulin is strongly recommended [93].

In conclusion, hyperglycemia is one of the most common and serious secondary complications of severe TBI. Future studies are needed to explore an optimal and safe glycemic target range, to facilitate critical care of blood glucose control and to further research the relationship between brain cell metabolism and hyperglycemia after severe TBI.

## References

[1] Wong VS, Langley B (2016). Epigenetic changes following traumatic brain injury and their implications for outcome, recovery and therapy. Neurosci Lett.

[2] Roozenbeek B, Maas AI, Menon DK (2013). Changing patterns in the epidemiology of traumatic brain injury. Nat Rev Neurol.

[3] Unterberg AW, Stover J, Kress B, Kiening KL (2004). Edema and brain trauma. Neuroscience.

[4] Myers RB, Lazaridis C, Jermaine CM, Robertson CS, Rusin CG (2016). Predicting Intracranial Pressure and Brain Tissue Oxygen Crises in Patients With Severe Traumatic Brain Injury. Crit Care Med.

[5] Chiu CC, Liao YE, Yang LY, Wang JY, Tweedie D, Karnati HK, Greig NH, Wang JY (2016). Neuroinflammation in animal models of traumatic brain injury. J Neurosci Methods.

[6] Murugan M, Santhakumar V, Kannurpatti SS (2016). Facilitating Mitochondrial Calcium Uptake Improves Activation-Induced Cerebral Blood Flow and Behavior after mTBI. Front Syst Neurosci.

[7] Reznik M, Saeed Y, Shutter L (2016). Teaching NeuroImages: Severe vasospasm in traumatic brain injury. Neurology.

[8] Chesnut RM (1995). Secondary brain insults after head injury: clinical perspectives. New Horiz.

[9] Siesjo BK (1993). Basic mechanisms of traumatic brain damage. Ann Emerg Med.

[10] Jeremitsky E, Omert L, Dunham CM, Protetch J, Rodriguez A (2003). Harbingers of poor outcome the day after severe brain injury: hypothermia, hypoxia, and hypoperfusion. J Trauma.

[11] Rovlias A, Kotsou S (2000). The influence of hyperglycemia on neurological outcome in patients with severe head injury. Neurosurgery.

[12] Cochran A, Scaife ER, Hansen KW, Downey EC (2003). Hyperglycemia and outcomes from pediatric traumatic brain injury. J Trauma.

[13] Lanzino G (2005). Plasma glucose levels and outcome after aneurysmal subarachnoid hemorrhage. J Neurosurg.

[14] Passero S, Ciacci G, Ulivelli M (2003). The influence of diabetes and hyperglycemia on clinical course after intracerebral hemorrhage. Neurology.

[15] Guo X, Li H, Zhang Z, Li S, Zhang L, Zhang J, Han G (2015). Hyperglycemia and Mortality Risk in Patients with Primary Intracerebral Hemorrhage: A Meta-Analysis. Mol Neurobiol.

[16] Falkowska A, Gutowska I, Goschorska M, Nowacki P, Chlubek D, Baranowska-Bosiacka I (2015). Energy Metabolism of the Brain, Including the Cooperation between Astrocytes and Neurons, Especially in the Context of Glycogen Metabolism. Int J Mol Sci.

[17] Simpson IA, Carruthers A, Vannucci SJ (2007). Supply and demand in cerebral energy metabolism: the role of nutrient transporters. J Cereb Blood Flow Metab.

[18] Guemes M, Rahman SA, Hussain K (2015). What is a normal blood glucose?. Arch Dis Child.

[19] Hall CN, Klein-Flugge MC, Howarth C, Attwell D (2012). Oxidative phosphorylation, not glycolysis, powers presynaptic and postsynaptic mechanisms underlying brain information processing. J Neurosci.

[20] Petit JM, Magistretti PJ (2015). Regulation of neuron-astrocyte metabolic coupling across the sleep-wake cycle. Neuroscience.

[21] Hertz L, Peng L, Dienel GA (2007). Energy metabolism in astrocytes: high rate of oxidative metabolism and spatiotemporal dependence on glycolysis/glycogenolysis. J Cereb Blood Flow Metab.

[22] Pellerin L (2008). Brain energetics (thought needs food). Curr Opin Clin Nutr Metab Care.

[23] Pellerin L, Magistretti PJ (2012). Sweet sixteen for ANLS. J Cereb Blood Flow Metab.

[24] Gandhi GK, Cruz NF, Ball KK, Dienel GA (2009). Astrocytes are poised for lactate trafficking and release from activated brain and for supply of glucose to neurons. J Neurochem.

[25] Heni M, Kullmann S, Preissl H, Fritsche A, Haring HU (2015). Impaired insulin action in the human brain: causes and metabolic consequences. Nat Rev Endocrinol.

[26] Kuo T, McQueen A, Chen TC, Wang JC (2015). Regulation of Glucose Homeostasis by Glucocorticoids. Adv Exp Med Biol.

[27] Alexiou GA, Lianos G, Fotakopoulos G, Michos E, Pachatouridis D, Voulgaris S (2014). Admission glucose and coagulopathy occurrence in patients with traumatic brain injury. Brain Inj.

[28] Chong SL, Harjanto S, Testoni D, Ng ZM, Low CY, Lee KP, Lee JH (2015). Early Hyperglycemia in Pediatric Traumatic Brain Injury Predicts for Mortality, Prolonged Duration of Mechanical Ventilation, and Intensive Care Stay. Int J Endocrinol.

[29] Bosarge PL, Shoultz TH, Griffin RL, Kerby JD (2015). Stress-induced hyperglycemia is associated with higher mortality in severe traumatic brain injury. J Trauma Acute Care Surg.

[30] Kerby JD, Griffin RL, MacLennan P, Rue LW (2012). Stress-induced hyperglycemia, not diabetic hyperglycemia, is associated with higher mortality in trauma. Ann Surg.

[31] Harp JB, Yancopoulos GD, Gromada J (2016). Glucagon Orchestrates Stress-induced Hyperglycemia. Diabetes Obes Metab.

[32] inKoshita K (2016). Traumatic brain injury: pathophysiology for neurocritical care. J Intensive Care.

[33] Strommer L, Wickbom M, Wang F, Herrington MK, Ostenson CG, Arnelo U, Permert J (2002). Early impairment of insulin secretion in rats after surgical trauma. Eur J Endocrinol.

[34] Gearhart MM, Parbhoo SK (2006). Hyperglycemia in the critically ill patient. AACN Clin Issues.

[35] Weaver LC, Bao F, Dekaban GA, Hryciw T, Shultz SR, Cain DP, Brown A (2015). CD11d integrin blockade reduces the systemic inflammatory response syndrome after traumatic brain injury in rats. Exp Neurol.

[36] Ruan H, Hacohen N, Golub TR, Van Parijs L, Lodish HF (2002). Tumor necrosis factor-alpha suppresses adipocyte-specific genes and activates expression of preadipocyte genes in 3T3-L1 adipocytes: nuclear factor-kappaB activation by TNF-alpha is obligatory. Diabetes.

[37] Gadek-Michalska A, Tadeusz J, Rachwalska P, Bugajski J (2013). Cytokines, prostaglandins and nitric oxide in the regulation of stress-response systems. Pharmacol Rep.

[38] Ley EJ, Srour MK, Clond MA, Barnajian M, Tillou A, Mirocha J, Salim A (2011). Diabetic patients with traumatic brain injury: insulin deficiency is associated with increased mortality. J Trauma.

[39] Liou DZ, Singer MB, Barmparas G, Harada MY, Mirocha J, Bukur M, Salim A, Ley EJ (2015). Insulin-dependent diabetes and serious trauma. Eur J Trauma Emerg Surg.

[40] Gorska M (2002). Diabetes mellitus in the elderly. Przegl Lek.

[41] Dusick JR, Wang C, Cohan P, Swerdloff R, Kelly DF (2012). Pathophysiology of hypopituitarism in the setting of brain injury. Pituitary.

[42] Schwartz MW, Porte D (2005). Diabetes, obesity, and the brain. Science.

[43] Salehi F, Kovacs K, Scheithauer BW, Pfeifer EA, Cusimano M (2007). Histologic study of the human pituitary gland in acute traumatic brain injury. Brain Inj.

[44] Daniel PM, Prichard MM, Schurr PH (1958). Extent of the infarct in the anterior lobe of the human pituitary gland after stalk section. Lancet.

[45] Daniel PM, Prichard MM (1966). Observations on the vascular anatomy of the pituitary gland and its importance in pituitary function. Am Heart J.

[46] Molitch ME, Clemmons DR, Malozowski S, Merriam GR, Shalet SM, Vance ML, Stephens PA, Endocrine Society's Clinical Guidelines S (2006). Evaluation and treatment of adult growth hormone deficiency: an Endocrine Society Clinical Practice Guideline. J Clin Endocrinol Metab.

[47] Broshek DK, De Marco AP, Freeman JR (2015). A review of post-concussion syndrome and psychological factors associated with concussion. Brain Inj.

[48] Howe LL (2009). Giving context to post-deployment post-concussive-like symptoms: blast-related potential mild traumatic brain injury and comorbidities. Clin Neuropsychol.

[49] Seyed Saadat SM, Bidabadi E, Seyed Saadat SN, Mashouf M, Salamat F, Yousefzadeh S (2012). Association of persistent hyperglycemia with outcome of severe traumatic brain injury in pediatric population. Childs Nerv Syst.

[50] Salim A, Hadjizacharia P, Dubose J, Brown C, Inaba K, Chan LS, Margulies D (2009). Persistent hyperglycemia in severe traumatic brain injury: an independent predictor of outcome. Am Surg.

[51] Jeremitsky E, Omert LA, Dunham CM, Wilberger J, Rodriguez A (2005). The impact of hyperglycemia on patients with severe brain injury. J Trauma.

[52] Bavisetty S, Bavisetty S, McArthur DL, Dusick JR, Wang C, Cohan P, Boscardin WJ, Swerdloff R, Levin H, Chang DJ, Muizelaar JP, Kelly DF (2008). Chronic hypopituitarism after traumatic brain injury: risk assessment and relationship to outcome. Neurosurgery.

[53] Sorensen L, Siddall PJ, Trenell MI, Yue DK (2008). Differences in metabolites in pain-processing brain regions in patients with diabetes and painful neuropathy. Diabetes Care.

[54] Kim GS, Jung JE, Narasimhan P, Sakata H, Chan PH (2012). Induction of thioredoxin-interacting protein is mediated by oxidative stress, calcium, and glucose after brain injury in mice. Neurobiol Dis.

[55] De Salles AA, Muizelaar JP, Young HF (1987). Hyperglycemia, cerebrospinal fluid lactic acidosis, and cerebral blood flow in severely head-injured patients. Neurosurgery.

[56] Palmer AM, Marion DW, Botscheller ML, Swedlow PE, Styren SD, DeKosky ST (1993). Traumatic brain injury-induced excitotoxicity assessed in a controlled cortical impact model. J Neurochem.

[57] Bullock R, Zauner A, Woodward JJ, Myseros J, Choi SC, Ward JD, Marmarou A, Young HF (1998). Factors affecting excitatory amino acid release following severe human head injury. J Neurosurg.

[58] Orrenius S, Zhivotovsky B, Nicotera P (2003). Regulation of cell death: the calcium-apoptosis link. Nat Rev Mol Cell Biol.

[59] Nishizawa Y (2001). Glutamate release and neuronal damage in ischemia. Life Sci.

[60] Rao VL, Baskaya MK, Dogan A, Rothstein JD, Dempsey RJ (1998). Traumatic brain injury down-regulates glial glutamate transporter (GLT-1 and GLAST) proteins in rat brain. J Neurochem.

[61] Allen NJ, Karadottir R, Attwell D (2004). Reversal or reduction of glutamate and GABA transport in CNS pathology and therapy. Pflugers Arch.

[62] Lucas DR, Newhouse JP (1957). The toxic effect of sodium L-glutamate on the inner layers of the retina. AMA Arch Ophthalmol.

[63] Rovegno M, Soto PA, Saez JC, von Bernhardi R (2012). Biological mechanisms involved in the spread of traumatic brain damage. Med Intensiva.

[64] Wieloch T, Nikolich K (2006). Mechanisms of neural plasticity following brain injury. Curr Opin Neurobiol.

[65] Kumar A, Loane DJ (2012). Neuroinflammation after traumatic brain injury: opportunities for therapeutic intervention. Brain Behav Immun.

[66] Laubertova L, Konarikova K, Gbelcova H, Durackova Z, Zitnanova I (2015). Effect of walnut oil on hyperglycemia-induced oxidative stress and pro-inflammatory cytokines production. Eur J Nutr.

[67] Pytel P, Alexander JJ (2009). Pathogenesis of septic encephalopathy. Curr Opin Neurol.

[68] Morganti-Kossmann MC, Rancan M, Otto VI, Stahel PF, Kossmann T (2001). Role of cerebral inflammation after traumatic brain injury: a revisited concept. Shock.

[69] Diringer MN, Scalfani MT, Zazulia AR, Videen TO, Dhar R, Powers WJ (2012). Effect of mannitol on cerebral blood volume in patients with head injury. Neurosurgery.

[70] Chesnut RM (2007). Care of central nervous system injuries. Surg Clin North Am.

[71] Alluri H, Wiggins-Dohlvik K, Davis ML, Huang JH, Tharakan B (2015). Blood-brain barrier dysfunction following traumatic brain injury. Metab Brain Dis.

[72] Fehnel CR, Wendell LC, Potter NS, Klinge P, Thompson BB (2014). Severe cerebral vasospasm after traumatic brain injury. R I Med J.

[73] Haddad SH, Arabi YM (2012). Critical care management of severe traumatic brain injury in adults. Scand J Trauma Resusc Emerg Med.

[74] Rapp RP, Young B, Twyman D, Bivins BA, Haack D, Tibbs PA, Bean JR (1983). The favorable effect of early parenteral feeding on survival in head-injured patients. J Neurosurg.

[75] Heyland DK, MacDonald S, Keefe L, Drover JW (1998). Total parenteral nutrition in the critically ill patient: a meta-analysis. Jama.

[76] Bilotta F, Rosa G (2012). Glycemia management in critical care patients. World J Diabetes.

[77] Oddo M, Schmidt JM, Mayer SA, Chiolero RL (2008). Glucose control after severe brain injury. Curr Opin Clin Nutr Metab Care.

[78] Khoury W, Klausner JM, Ben-Abraham R, Szold O (2004). Glucose control by insulin for critically ill surgical patients. J Trauma.

[79] Meier R, Bechir M, Ludwig S, Sommerfeld J, Keel M, Steiger P, Stocker R, Stover JF (2008). Differential temporal profile of lowered blood glucose levels (3.5 to 6.5 mmol/l versus 5 to 8 mmol/l) in patients with severe traumatic brain injury. Crit Care.

[80] van den Berghe G, Wouters P, Weekers F, Verwaest C, Bruyninckx F, Schetz M, Vlasselaers D, Ferdinande P, Lauwers P, Bouillon R (2001). Intensive insulin therapy in critically ill patients. N Engl J Med.

[81] Finfer S, Chittock DR, Su SY, Blair D, Foster D, Dhingra V, Bellomo R, Cook D, Dodek P, Henderson WR, Hebert PC, Heritier S, Investigators N-SS (2009). Intensive versus conventional glucose control in critically ill patients. N Engl J Med.

[82] Oddo M, Schmidt JM, Carrera E, Badjatia N, Connolly ES, Presciutti M, Ostapkovich ND, Levine JM, Le Roux P, Mayer SA (2008). Impact of tight glycemic control on cerebral glucose metabolism after severe brain injury: a microdialysis study. Crit Care Med.

[83] Vespa P, Boonyaputthikul R, McArthur DL, Miller C, Etchepare M, Bergsneider M, Glenn T, Martin N, Hovda D (2006). Intensive insulin therapy reduces microdialysis glucose values without altering glucose utilization or improving the lactate/pyruvate ratio after traumatic brain injury. Crit Care Med.

[84] Badjatia N, Vespa P (2014). Monitoring nutrition and glucose in acute brain injury. Neurocrit Care.

[85] Zhi D, Zhang S, Lin X (2003). Study on therapeutic mechanism and clinical effect of mild hypothermia in patients with severe head injury. Surg Neurol.

[86] Zhao QJ, Zhang XG, Wang LX (2011). Mild hypothermia therapy reduces blood glucose and lactate and improves neurologic outcomes in patients with severe traumatic brain injury. J Crit Care.

[87] Zhang H, Wang X, Li Y, Du R, Xu E, Dong L, Wang X, Yan Z, Pang L, Wei M, She L (2014). Naloxone for severe traumatic brain injury: a meta-analysis. PloS one.

[88] Zink BJ, Schultz CH, Stern SA, Mertz M, Wang X, Johnston P, Keep RF (2001). Effects of ethanol and naltrexone in a model of traumatic brain injury with hemorrhagic shock. Alcohol Clin Exp Res.

[89] Boone MD, Oren-Grinberg A, Robinson TM, Chen CC, Kasper EM (2015). Mannitol or hypertonic saline in the setting of traumatic brain injury: What have we learned?. Surg Neurol Int.

[90] Burgess S, Abu-Laban RB, Slavik RS, Vu EN, Zed PJ (2016). A Systematic Review of Randomized Controlled Trials Comparing Hypertonic Sodium Solutions and Mannitol for Traumatic Brain Injury: Implications for Emergency Department Management. Ann Pharmacother.

[91] Bellander BM, Cantais E, Enblad P, Hutchinson P, Nordstrom CH, Robertson C, Sahuquillo J, Smith M, Stocchetti N, Ungerstedt U, Unterberg A, Olsen NV (2004). Consensus meeting on microdialysis in neurointensive care. Intensive Care Med.

[92] Hillered L, Persson L, Nilsson P, Ronne-Engstrom E, Enblad P (2006). Continuous monitoring of cerebral metabolism in traumatic brain injury: a focus on cerebral microdialysis. Curr Opin Crit Care.

[93] Vriesendorp TM, DeVries JH, Holleman F, Dzoljic M, Hoekstra JB (2005). The use of two continuous glucose sensors during and after surgery. Diabetes Technol Ther.

